# Frailty is a useful predictive marker of postoperative complications after pancreaticoduodenectomy

**DOI:** 10.1186/s12957-020-01969-7

**Published:** 2020-08-03

**Authors:** Yutaka Nakano, Yuki Hirata, Tatsuya Shimogawara, Toru Yamada, Koki Mihara, Ryo Nishiyama, Shin Nishiya, Hideki Taniguchi, Tomohisa Egawa

**Affiliations:** 1 Department of Surgery, Saiseikai Yokohamashi Tobu Hospital, 3-6-1 Shimosueyoshi, Tsurumi-ku, Yokohama, Kanagawa 230-0012 Japan; 2 Department of Patients Support Center, Saiseikai Yokohamashi Tobu Hospital, 3-6-1 Shimosueyoshi, Tsurumi-ku, Yokohama, Kanagawa 230-0012 Japan

**Keywords:** Frailty, Sarcopenia, Pancreaticoduodenectomy, Postoperative complications, Clinically relevant postoperative pancreatic fistula

## Abstract

**Background:**

Frailty results in a high risk for disability, hospitalization, and mortality. This study aimed to investigate perioperative details of frail patients who underwent pancreatectomy and whether frailty can be a predictive factor of postoperative complications, especially of clinically relevant postoperative pancreatic fistula (CR-POPF).

**Methods:**

This retrospective study included patients who underwent pancreatectomy in our hospital between August 2016 and March 2019. The patients were divided into frail and pre-/non-frail groups. The diagnostic criteria were based on the Japanese version of the Cardiovascular Health Study.

**Results:**

Of 93 patients, 11 (11.8%) and 82 (88.2%) were frail and pre-/non-frail patients, with median ages of 82 and 72 years, respectively (*p* = 0.041). Postoperative complications (Clavien-Dindo ≧ IIIa) were found in 8 and 32 patients (*p* = 0.034), CR-POPF in 3 and 13 patients (*p* = 0.346), and postoperative hospital stays were 21 and 17 days (*p* = 0.041), respectively. On multivariate analysis, frailty was an independent predictive factor (odds ratio [OR] 5.604, 95.0% confidence interval [CI] 1.002-30.734; *p* = 0.047) of postoperative complications (Clavien-Dindo ≧ IIIa) after pancreaticoduodenectomy. On multivariate analysis, a soft pancreas (OR 5.696, 95.0% CI 1.142-28.149; *p* = 0.034) was an independent and significant predictive factor of CR-POPF after pancreaticoduodenectomy.

**Conclusions:**

Frailty may be a useful predictive factor of postoperative complications in patients undergoing pancreaticoduodenectomy.

## Background

Frailty has become the center of attention in the geriatric field because it is considered to result in a high risk for falls, disability, hospitalization, and mortality [1]. Pancreatectomy remains one of the most life-threatening abdominal surgeries associated with mortality [2]. The proportion of the elderly population has increased not only in other countries but also in Japan [3], which in turn has increased the number of elderly patients undergoing pancreatectomy. Many pancreatectomies have been performed for malignancy, and compared with younger patients, elderly patients are at a risk for increased morbidity and mortality [4]. The risk of frailty is higher in the elderly population, and frailty predicts severe complications and mortality after pancreatectomies [5]. The safety of pancreatectomy performed in community cancer centers is similar to that performed in any academic center or university hospital [6]. However, accurate evaluation and reduction of preoperative risk in the elderly population are essential, especially among community cancer centers.

Sarcopenia can be considered one of the main physical drivers of frailty or even a precursor state [7], and it has been considered one of the risk stratification tools to better identify potentially high-risk surgical patients [8]. A systematic review and meta-analysis [9] has reported an increase in the duration of inpatient hospital stay of sarcopenia patients. Several reports [5, 10, 11] have reported frailty as an important independent predictor of outcomes after pancreatic surgery; however, to the best of our knowledge, the relationship between frail patients and pre-/non-frail patients or that between frailty and sarcopenia has not been extensively studied.

Thus, in this study, our primary aim was to evaluate the relationship between frailty and sarcopenia and investigate the clinicopathological characteristics of frail patients who had pancreatic resection, focusing on perioperative short-term outcomes, such as postoperative complications, especially postoperative pancreatic fistula (POPF). Moreover, our secondary aim was to evaluate whether frailty can be a predictive factor of postoperative complications (Clavien-Dindo classification ≥ IIIa) (CD ≥ IIIa) or clinically relevant postoperative pancreatic fistula (CR-POPF; grades B/C POPF).

## Methods

### Patients

Data of patients who underwent intended curative pancreatectomy (distal pancreatectomy and pancreaticoduodenectomy) at our institution between August 2016 and March 2019, were retrospectively reviewed. We excluded patients who were made to change surgical procedure to total pancreatectomy. This retrospective observational study used the “opt-out” method of our hospital. The study was approved by the Ethics Committee of Saiseikai Yokohamashi Tobu Hospital (ethical approval number: 20190032). Research was conducted in accordance with the Declaration of Helsinki 1975.

### Preoperative assessment in patient support center

Since 2016, our hospital has established a patient support center where various conditions of preoperative patients have been assessed by anesthesiologists, nurses, pharmacists, registered dietitians, and dental hygienists from the viewpoint of enhanced recovery after surgery program [12]. In the center, demographic and clinical variables such as age, sex, body mass index, presence or absence of smoking (current and former) and alcohol intake history, past medical history, and medicines used (especially antithrombotic drugs) were assessed. Moreover, preoperative laboratory data (serum albumin, lymphocyte, total cholesterol, and hemoglobin levels, prognostic nutritional index [13], and controlling nutritional status score [14]) were evaluated.

### Definition of sarcopenia and frailty

In the patient support center, we asked patients regarding their health condition, such as weight loss, physical activity, and walking speed, and measured grip strength. Multi-frequency bioelectrical impedance analysis (InBody 770; Biospace, Tokyo, Japan) was performed to assess preoperative skeletal muscle mass. In this study, we defined sarcopenia according to the criteria of the Asian Working Group for Sarcopenia [15], and to diagnose frailty, we used the Japanese version of the cardiovascular health study (J-CHS) criteria, which was similar to previous studies that used the CHS criteria to identify frailty [16]. The J-CHS criteria comprise five items and questions—(i) shrinking: have you lost ≥ 2 kg in the past 6 months?; (ii) low activity: do you engage in moderate levels of physical exercise or sports aimed at health?; and do you engage in low levels of physical exercise aimed at health?; (iii) exhaustion: in the past 2 weeks, have you felt tired without a reason? (iv) weakness: grip strength < 26 kg in men or 18 kg in women; and (v) slowness: gait speed < 1.0 m/s. Frailty, pre-frailty, and non-frailty are defined as having 3–5, 1–2, and 0 components, respectively. To investigate the relationship between frailty and sarcopenia, we adopted the J-CHS criteria, which included similar items to the criteria of sarcopenia, such as grip strength and walking time as weakness and slowness, respectively.

### Surgery and postoperative assessment

**Fig. 1:**
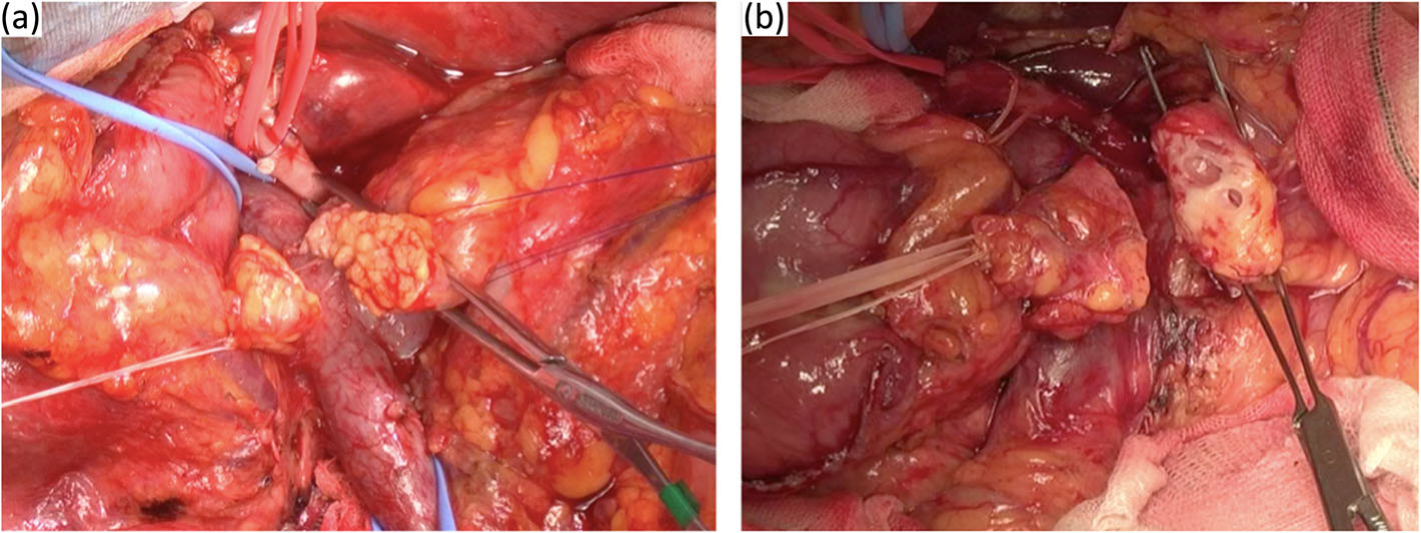
Cut margin of the soft pancreas (**a**) and hard pancreas (**b**) during pancreaticoduodenectomy

Surgery included pancreaticoduodenectomy or distal pancreatectomy for malignant and benign tumors. D2 lymph node dissection was performed in all cancer patients. To evaluate the pancreas intraoperatively as soft or hard, surgeons judged the pancreas status subjectively (Fig. 1). Postoperative complications (e.g., POPF, bile leakage, fluid collection, intra-abdominal bleeding, and delayed gastric emptying) were evaluated according to the Clavien-Dindo classification. In this study, we especially focused on CR-POPF according to 2016 the International Study Group of Pancreatic Fistula classification [17].

### Statistical analyses

Patients were divided into the frail group and the pre-/non-frail group based on their frailty status defined according to the J-CHS criteria. The clinicopathological characteristics between the frail group and the pre-/non-frail group and between the frail group and the sarcopenia group were evaluated. Categorical variables were compared using the chi-square test or Fisher’s exact test, and continuous variables were compared using the Mann-Whitney *U* test. Variables that were significant in the univariate analysis (*p* < 0.10) were included in the logistic regression analyses to identify independent predictive factors of postoperative complications (CD ≥ IIIa) and CR-POPF. We analyzed independent predictive factors of not only postoperative complications (CD ≥ IIIa) but also CR-POPF in the pancreaticoduodenectomy group and distal pancreatectomy group separately because the proposed mechanism of pancreatic fistula is different between pancreaticoduodenectomy and distal pancreatectomy [18].

All statistical analyses were performed using Statistical Package for the Social Sciences for Macintosh, software version 25.0 (IBM Corp., Armonk, NY, USA). *P* < 0.05 was considered statistically significant.

## Results

### Patient characteristics in the frail, pre-/non-frail, frail, and sarcopenia groups

In total, 95 patients underwent curative pancreatectomy between August 2016 and March 2019. Of them, two patients had undergone total pancreatectomy and one patient had schizophrenia; as we could not perform accurate evaluation at the patient support center, they were excluded. Therefore, 93 patients were enrolled for the analysis. Of the 93 patients, 11 (11.8%) patients were included in the frail group and 82 (88.2%) patients were included in the pre-/non-frail group. The overall patient characteristics and demographic and clinical characteristics of the frail and pre-/non-frail group are listed in Table 1. All frail patients had sarcopenia; hence, we compared frail patients with sarcopenic patients who did not satisfy the J-CHS criteria in terms of their clinical characteristics, including details of postoperative complications with CD ≥ IIIa (Table 2). The clinical characteristic details of the 11 frail patients are shown in Table 3.

**Table 1:** Demographic and clinical characteristics or all patients and between frail and pre-/non-frail patients

	Total (*N* = 93)	Frail (*N* = 11)	Pre-/non-frail (*N* = 82)	*p* value
Age (years)	72 (27-88)	82 (69-88)	72 (27-85)	0.041
Sex (male/female)	57/36	4/7	53/29	0.071
Body mass index (kg/m^2^)	21.7 (14.2-33.0)	19.7 (14.5-24.9)	22.0 (14.2-33.0)	0.242
Smoking (current and former)	45 (48.4%)	2 (18.2%)	43 (52.4%)	0.033
Alcohol	6 (6.5%)	0 (0.0%)	6 (7.1%)	0.350
Diabetes mellitus	23 (25.9%)	5 (45.5%)	18 (22.0%)	0.090
Antithrombotic drugs	21 (22.6%)	5 (45.5%)	16 (19.5%)	0.053
Grip strength (kg)	27.0 (10.0-48.0)	14.6 (10.0-22.8)	27.4 (13.1-48.0)	0.002
Skeletal muscle index (kg/m^2^)	6.7 (4.0-8.9)	4.7 (4.0-5.7)	6.8 (4.7-8.9)	0.063
Sarcopenia	37 (39.8%)	11 (100.0%)	26 (31.7%)	< 0.001
Disease				0.539
Pancreatic cancer	46 (49.5%)	6 (54.5%)	40 (48.8%)	
Bile duct cancer (including papilla of Vater)	20 (21.5%)	4 (36.4%)	16 (19.5%)	
Intraductal papillary mucinous neoplasm	9 (9.7%)	0 (0.0%)	9 (11.0%)	
Pancreatic neuroendocrine tumor	6 (6.5%)	0 (0.0%)	6 (7.3%)	
Benign tumor	5 (5.4%)	0 (0.0%)	5 (6.1%)	
Others	7 (7.5%)	1 (9.1%)	6 (7.3%)	
Surgical procedure				0.488
Pancreatoduodenectomy	68 (73.1%)	9 (81.8%)	59 (72.0%)	
Distal pancreatectomy	25 (26.9%)	2 (18.2%)	23 (28.0%)	
Soft pancreas	62 (66.7%)	7 (63.6%)	55 (67.1%)	0.820
Albumin (g/l)	4.0 (2.6-4.9)	3.3 (2.6-4.0)	4.0 (2.7-4.9)	0.009
Lymphocyte (×10^3^/μl)	1551 (530-3724)	1242 (530-2124)	1568 (540-3724)	0.546
Total cholesterol (mg/dl)	198 (72-335)	137 (72-230)	200 (89-335)	0.156
Hemoglobin (g/dl)	12.9 (8.4-19.6)	11.3 (9.4-14.0)	13.1 (8.4-19.6)	0.059
Prognostic nutritional index	48.2 (32.1-62.6)	36.2 (32.3-48.2)	49.0 (32.1-62.6)	0.002
Controlling nutritional status				< 0.001
0〜1 or 2〜4	84 (90.3%)	4 (36.4%)	80 (97.6%)	
5〜8 or 8〜	9 (9.7%)	7 (63.6%)	2 (2.4%)	
Operative time (min)	514 (206-874)	563 (228-874)	503 (206-872)	0.162
Blood loss (g)	685 (75-5671)	985 (223-2703)	662 (75-5671)	0.186
Intraoperative transfusion	20 (21.5%)	6 (54.5%)	14 (17.1%)	0.005
Clavien-Dindo classification ≧ IIIa	40 (43.0%)	8 (72.7%)	32 (39.0%)	0.034
Clinically relevant postoperative pancreatic fistula	16 (17.2%)	3 (27.3%)	13 (15.9%)	0.346
Postoperative hospital stay (day)	18 (7-431)	21 (14-83)	17 (7-431)	0.041
Postoperative 30-day mortality	1 (1.1%)	1 (9.1%)	0 (0.0%)	0.006
Postoperative 90-day mortality	3 (3.2%)	3 (27.3%)	0 (0.0%)	< 0.001

Values in median

**Table 2:** Demographic and clinical characteristics between frail and sarcopenia

	Sarcopenia (*N* = 37)		*p* value
	Frail (*N* = 11, 29.7%)	Not frail^*^ (*N* = 26, 70.3%)	
Age (years)	82 (69-88)	76 (59-85)	0.026
Sex (male/female)	4/7	13/13	0.447
Medical history			
Diabetes mellitus	5 (45.5%)	4 (15.4%)	0.051
Cardiac valvular disease	2 (18.2%)	0 (0.0%)	0.025
Myocardial infarction	2 (18.2%)	2 (7.7%)	0.348
Chronic pulmonary disease or pneumonia	4 (36.4%)	0 (0.0%)	0.001
Hypertension requiring medication	3 (27.3%)	7 (26.9%)	0.546
Cerebrovascular accident	3 (27.3%)	0 (0.0%)	0.004
Albumin (g/l)	3.3 (2.6-4.0)	3.7 (3.1-4.6)	0.218
Prognostic nutritional index	36.2 (32.3-48.2)	47.1 (38.8-58.9)	0.540
Controlling nutritional status			<0.001
0〜1 or 2〜4	4 (36.4%)	26 (100%)	
5〜8 or 8〜	7 (63.6%)	0 (0.0%)	
Operative time (min)	563 (228-874)	497 (206-753)	0.122
Blood loss (g)	985 (223-2703)	646 (75-2613)	0.122
Intraoperative transfusion	6 (54.5%)	5 (19.2%)	0.032
Clavien-Dindo classification ≧ IIIa	8 (72.7%)	10 (38.5%)	0.057
Clinically relevant postoperative pancreatic fistula	3 (27.3%)	7 (26.9%)	0.983
Intra-abdominal abscess	1 (9.1%)	2 (7.7%)	0.887
Bile leakage	0 (0.0%)	1 (3.8%)	0.510
Wound dehiscence	1 (9.1%)	1 (3.8%)	0.519
Organ/space surgical site infection	1 (9.1%)	0 (0.0%)	0.119
Respiratory failure	3 (27.3%)	0 (0.0%)	0.005
Postoperative hospital stay (day)	21 (14-83)	18 (8-431)	0.408
Postoperative 30-day mortality	1 (9.1%)	0 (0.0%)	0.119
Postoperative 90-day mortality	3 (27.3%)	0 (0.0%)	0.005

Values in median

*Not frail: sarcopenia patients who were not satisfied the J-CHS criteria

**Table 3:** Details of the clinical characteristics of frail patients

No	Age (years)	Sex	Medical history	Disease	Surgical procedure	Postoperative complications (Clavien-Dindo classification ≧ IIIa)	30-day postoperative mortality (cause of death)	90-day postoperative mortality (cause of death)
1	86	F	AS, heart pacemaker	PC	DP	Pancreatic fistula (grade B)	No	No
2	88	F	AS, DM	PC	PD		No	No
3	88	F	CI, DM, PE	BC	PD	Pancreatic fistula (grade C), pseudoaneurysm s/o, pylethrombosis, melena	No	Yes (acute respiratory failure)
4	81	M	Bronchiectasis	PC	PD	Bacterial pneumonia, ARDS, wound dehiscence	No	Yes (acute respiratory failure, DIC)
5	72	M	Gastric caner	Remnant gastric cancer	PD	Esophagojejunostomy leakage, pancreatic fistula (grade C), aspiration pneumonia	Yes (acute respiratory failure, septic shock)	No
6	69	M	CI, MI	BC	PD	Intra-abdominal abscess	No	No
7	82	M	CI, MI, PAD	BC	PD	Paralytic ileus	No	No
8	79	F	Gastric ulcer	BC	PD	Anastomotic bleeding of gastrojejunostomy	No	No
9	85	F	Pulmonary tuberculosis	PC	PD		No	No
10	87	F	DM	PC	DP	Organ/space surgical site infection	No	No
11	81	F	Duodenal ulcer	PC	PD		No	No

Abbreviations: *ARDS* acute respiratory distress syndrome, *AS* aortic stenosis, *BC* bill duct cancer, *CI* cerebral infarction; *DIC* disseminated intravascular coagulation, *DM* diabetes mellitus, *DP* distal pancreatectomy, *F* female, *M* male, *MI* myocardial infarction, *PAD* peripheral arterial disease, *PC* pancreatic cancer, *PD* pancreaticoduodenectomy, *PE* pulmonary embolism

### Predictive factors for postoperative complications (CD ≥ IIIa) and CR-POPF after pancreaticoduodenectomy and distal pancreatectomy

Predictive factors associated with postoperative complications (CD ≥ IIIa) and CR-POPF in the pancreaticoduodenectomy (*N* = 68, 73.1%) and distal pancreatectomy (*N* = 25, 26.9%) groups are shown in Tables 4 and 5. In multivariate analysis, frailty (odds ratio [OR] 5.604, 95.0% confidence interval [CI] 1.022-30.734; *p* = 0.047) was the only independent and significant predictive factor of postoperative complications (CD ≥ IIIa) in the pancreaticoduodenectomy group. In contrast, soft pancreas (OR 5.696, 95.0% CI 1.142-28.149; *p* = 0.034) an independent and significant predictive factor of CR-POPF in the pancreaticoduodenectomy group. In this study, both univariate and multivariate analyses did not reveal predictive factors of postoperative complications (CD ≥ IIIa) and CR-POPF in the distal pancreatectomy group.

**Table 4:** Univariate and multivariate analyses of predictive factors of postoperative complications with CD ≧ IIIa and CR-POPF in the pancreaticoduodenectomy group (*N* = 68)

Factor	Postoperative complications with CD ≧ IIIa						CR-POPF					
	Univariate			Multivariate			Univariate			Multivariate		
	*p* value	Odds ratio	95% Cl for Exp (B)	*p* value	Odds ratio	95% Cl for Exp (B)	*p* value	Odds ratio	95% Cl for Exp (B)	*p* value	Odds ratio	95% Cl for Exp (B)
Age (years)	0.62	1.036	0.986-1.089				0.339	1.031	0.968-1.099			
Sex (female/male)	0.669	1.240	0.463-3.323				0.313	0.543	0.166-1.779			
BMI (kg/m2)	0.771	0.997	0.984-1.011				0.331	1.007	0.993-1.022			
Smoking	0.672	1.230	0.442-3.210				0.818	0.871	0.266-2.848			
Alcohol	0.410	0.378	0.037-3.828				0.999	0.000	0.000			
Disease	0.811	0.964	0.715-1.301				0.160	1.274	0.909-1.787			
Diabetes mellitus	0.209	2.114	0.657-6.801				0.437	0.526	0.104-2.661			
Antithrombotic drugs	0.210	2.041	0.669-6.225				0.729	1.262	0.338-4.707			
Operative time (min)	0.809	0.999	0.995-1.004				0.095	0.995	0.998-1.001	0.184	0.996	0.989-1.002
Blood loss (g)	0.980	1.000	0.999-1.001				0.554	1.000	0.999-1.001			
Intraoperative transfusion	0.720	0.822	0.282-2.369				0.544	0.648	0.159-2.637			
Soft pancreas	0.074	2.471	0.916-6.666	0.065	2.656	0.941-7.500	0.021	6.462	1.319-31.663	0.034	5.696	1.142-28.149
Albumin (g/l)	0.287	0.603	0.237-1.533				0.954	0.968	0.314-2.980			
Lymphocyte (× 10^3^/μl)	0.321	1.000	1.000-1.001				0.348	1.000	1.000-1.001			
Total cholesterol (mg/dl)	0.138	0.991	0.979-1.003				0.148	0.988	0.972-1.004			
Hemoglobin (g/dl)	0.533	1.046	0.907-1.207				0.701	0.968	0.821-1.141			
PNI	0.836	1.006	0.951-1.065				0.551	1.024	0.946-1.109			
CONUT score	0.483	0.779	0.388-1.565				0.162	0.504	0.193-1.317			
Sarcopenia	0.642	1.255	0.482-3.265				0.192	2.250	0.666-7.605			
Frail	0.054	5.104	0.975-26.713	0.047	5.604	1.022-30.734	0.896	1.119	0.266-6.092			

Abbreviations: *BMI* body mass index, *CD* Clavien-Dindo classification, *COUNT* controlling nutritional status, *CR-POPF* clinically relevant postoperative pancreatic fistula, *PNI* prognostic nutritional index

**Table 5:** Univariate and multivariate analyses of predictive factors of postoperative complications with CD ≧ IIIa and CR-POPF in the distal pancreatectomy group (*N* = 25)

Factor	Postoperative complications with CD ≧ IIIa						CR-POPF					
	Univariate			Multivariate			Univariate			Multivariate		
	*p* value	Odds ratio	95% Cl for Exp (B)	*p* value	Odds ratio	95% Cl for Exp (B)	*p* value	Odds ratio	95% Cl for Exp (B)	*p* value	Odds ratio	95% Cl for Exp (B)
Age (years)	0.476	1.024	0.960-1.091				0.910	0.994	0.900-1.098			
Sex (female/male)	0.611	1.556	0.284-8.531				0.765	0.643	0.036-11.631			
BMI (kg/m2)	0.126	1.230	0.944-1.603				0.804	1.048	0.726-1.512			
Smoking	0.386	0.480	0.091-2.523				0.859	0.769	0.043-13.866			
Alcohol	0.999	0.000	0.000				0.081	21.000	0.686-642.982	0.997	0.000	0.000
Disease	0.796	0.928	0.527-1.634				0.298	1.563	0.673-3.629			
Diabetes mellitus	0.915	1.110	0.192-6.286				0.578	2.286	0.124-41.985			
Antithrombotic drugs	0.621	0.542	0.048-6.144				0.219	6.667	0.323-137.403			
Operative time (min)	0.868	1.001	0.992-1.010				0.865	1.001	0.986-1.017			
Blood loss (g)	0.471	1.001	0.999-1.002				0.305	1.001	0.999-1.004			
Intraoperative transfusion	1.000	0.000	0.000				1.000	0.000	0.000			
Soft pancreas	1.000	0.000	0.000				1.000	0.000	0.000			
Albumin (g/l)	0.058	0.037	0.001-1.121	0.366	0.108	0.001-13.492	0.709	0.388	0.003-55.874			
Lymphocyte (× 10^3^/μl)	0.492	0.999	0.998-1.001				0.099	1.002	1.000-1.004	0.997	1.020	0.000
Total cholesterol (mg/dl)	0.602	1.006	0.983-1.031				0.097	0.970	0.936-1.006	0.999	0.759	0.000
Hemoglobin (g/dl)	0.697	0.887	0.484-1.625				0.993	0.995	0.344-2.881			
PNI	0.084	0.794	0.612-1.031	0.559	0.901	0.634-1.279	0.311	1.189	0.851-1.661			
CONUT score	0.511	1.760	0.326-9.510				0.671	1.875	0.103-34.131			
Sarcopenia	0.529	2.000	0.231-17.338				0.219	6.667	0.323-137.403			
Frail	0.671	1.875	0.103-34.131				0.077	22.000	0.719-672.782	0.998	0.000	0.000

Abbreviations: *BMI* body mass index, *CD* Clavien-Dindo classification, *COUNT* controlling nutritional status, *CR-POPF* clinically relevant postoperative pancreatic fistula, *PNI* prognostic nutritional index

## Discussion

This study investigated not only clinical characteristics between frail and pre-/non-frail patients and frail patients with sarcopenic patients but also predictive factors related to postoperative complications and CR-POPF. In this study, frailty and soft pancreas were an independent and significant predictive factor of postoperative complications (CD ≥ IIIa) and CR-POPF after pancreaticoduodenectomy, respectively.

Many physicians often observe that some patients can withstand operational stress, while others cannot despite being of the same chronological age, and they judge instinctively and subjectively whether patients have the physiological reserve to endure operations and postoperative burdens. Although some older patients do not have such reserve to endure surgical stress [19], there are appropriate methods for evaluating older surgical patients. Our results demonstrate that frailty may be a useful predictive factor of postoperative complications in patients undergoing pancreatectomy and may become one of the risk stratification tools to better identify potentially high-risk surgical patients. Unlike sarcopenia, frailty represents not only the skeletal muscle mass and muscle function but also physical activity in daily living, weight loss, and social isolation [6]. Thus, frailty is considered a biologic syndrome of decreased reserve and resistance to stressors, resulting from cumulative decline across multiple physiologic systems and causing vulnerability to adverse outcomes [1]. Our findings suggest that frailty is a more effective predictor than sarcopenia to evaluate potentially high-risk surgical patients, even if these two conditions start to converge because of their close relationship with the aging process [6].

Several reports [9–11] have revealed that frailty is an important predictor of postoperative morbidity and mortality after pancreatectomy, which is consistent with our study results. These studies used the modified frailty index (mFI) to define frailty [20], while our study used the J-CHS criteria. The mFI is a simple frailty assessment tool mainly evaluated by the patient’s historical variables, such as history of myocardial infarction, previous coronary operation, chronic obstructive pulmonary disease, or pneumonia. In contrast, the J-CHS criteria are mainly comprise patients’ physical ability and conditions, such as shrinking, weakness, poor endurance, slowness, and low activity. Although it is important to focus on a patient’s historical variables, such as mFI, we aimed to investigate the relationship between frailty and sarcopenia, which is a progressive and generalized skeletal muscle disorder involving the accelerated loss of muscle mass and function. Thus, we adopted the J-CHS criteria, which included similar items to the criteria of sarcopenia, such as grip strength and walking time. Unlike these previous studies, our study focused on the relationship between frailty and sarcopenia. Table 2 shows that compared with sarcopenia patients who did not satisfied the J-CHS criteria, frail patients had pulmonary, neurologic, or cardiac medical histories and diabetes mellitus, which may influence postoperative morbidity and mortality after pancreatectomy. Moreover, frail patients had more postoperative complications with CD ≥ IIIa than sarcopenia (not frail) patients (*p* = 0.087). No difference in the occurrence frequency of CR-POPF was found between the two groups, but a significant difference was found in the occurrence frequency of respiratory failure (*p* = 0.030), which resulted in postoperative mortality in frail patients. Sarcopenia was a risk stratification tool to better identify potentially high-risk surgical patients [7], but frailty was also a useful predictive factor of postoperative complications and may be an effective risk stratification tool to identify these potentially high-risk surgical patients.

Our report also focused on CR-POPF, which was not discussed in previous reports [9–11]. CR-POPF remains one of the most life-threatening postoperative complications, and two frail patients in our study, who died within 90 days after pancreaticoduodenectomy, had CR-POPF. The direct cause of death of these patients was acute respiratory failure, which could have triggered uncontrollable CR-POPF. Frail patients may not have physiological reserve to endure postoperative life-threatening complications, such as CR-POPF. Several reports [21, 22] considered that the soft texture of the pancreatic parenchyma could contribute to the development of POPF after pancreaticoduodenectomy. A soft pancreas and a small-diameter pancreatic duct preserve exocrine function, which increases the secretion of pancreatic juice and pressure within the pancreaticoenteric lumen [22]; our findings were consistent with these findings. However, in our study, “soft” pancreas was subjectively judged by the surgeons. Moreover, “soft” or “hard” pancreas is associated with pancreatic tissue fibrosis, and several previous studies have attempted to quantify pancreatic fibrosis and have suggested that a pancreas with less fibrosis, more fatty tissues, and more acinar cells is at risk for POPF [23]. Fujita et al. [23] reported a useful approach for quantifying pancreatic tissue objectively by acoustic radiation force impulse imaging, and pancreatic tissue fibrosis was found to be correlated with the overall incidence of POPF. In contrast, POPF after distal pancreatectomy is due to functional distal obstruction by the sphincter of Oddi complex at the ampulla [24]. Our study did not reveal the predictive factor of CR-POPF after distal pancreatectomy; further studies should be performed to evaluate CR-POPF after distal pancreatectomy.

In frail patients undergoing surgery, surgeons should consider various interventions preoperatively, intraoperatively, or postoperatively to reduce postoperative complications. Nutritional status and frailty are interrelated [25]; hence, preoperative intervention for nutritional status may improve frail status. In two randomized double-blind studies [26, 27], both exercise and nutrition improved muscle mass, walking ability, and hematological parameters, possibly leading to the reversal of the frailty status. In these reports, resistance-type exercise training was effective in improving strength and physical performance in frail patients, and supplements were recommended during exercise training. This preoperative intervention is called “prehabilitation,” which is a collective term to describe preoperative interventions aimed at increasing the physiological reserve of patients prior to surgery. Prehabilitation programs variably include physical, psychological, and nutritional interventions and may reduce the incidence of postoperative complications, shorten hospital stay, and improve health-related quality of life [28]. Despite the lack of evidence of improved mortality and duration of hospital stay, various beneficial prehabilitation programs for frail surgical patients have been reported in a systematic review [28]. Thus, we should consider both exercise and nutritional intervention preoperatively. Conversely, early postoperative nutritional support helps reduce the risk of postoperative complications, especially postoperative early enteral nutrition, which improves the nutritional status and promotes functional recovery of the digestive system [29]. As one of the intraoperative interventions, Gilliland et al. [30] recommended that in pancreatic cancer patients with moderately decreased albumin levels (< 3.0 mg/dL) or weight loss > 5%, jejunostomy feeding tubes should be used intraoperatively to avoid postoperative undesirable patient outcomes associated with insufficient nutritional intervention. Moreover, to avoid postoperative complications, it may be useful to insert an enteral tube after a more invasive surgery, such as pancreaticoduodenectomy, as an early nutritional support for frail patients with poor nutritional status.

In this study, three patients died; the main cause of death was acute respiratory failure. Postoperative complications (CD ≥ IIIa) in these three patients varied; two of them had CR-POPF. Considering our results, frail patients undergoing pancreaticoduodenectomy should have preoperative prehabilitation, especially respiratory prehabilitation [31]. In a study by Varga JT [31], a respiratory prehabilitation program provided a positive effect on the cardiovascular system, metabolism, muscles, and lung mechanics, resulting in optimal functional condition and less postoperative complication. This prehabilitation was supposed to improve nutritional status, strength, physical performance, and frail status. We need to consider the duration of prehabilitation as a long-duration prehabilitation program may result in disease progression, especially in pancreatic cancer or bile duct cancer patients. If the preoperative frail status does not improve, pancreatectomy should be avoided in frail patients and other treatments, such as chemotherapy, radiotherapy, or chemoradiotherapy, should be considered. Surgery is a radical treatment, especially for pancreatic cancer and bile duct cancer. This problem is puzzling for many surgeons.

Consideration of frailty may be beneficial for the evaluation of operative risk and selection of patients.

This study has several limitations. First, this retrospective study as conducted on a very small scale compared with previous reports because of its single-institution setting; thus, future multi-institutional prospective research studies are needed. Second, although previous reports [22, 23] have revealed objective evaluation of pancreatic fibrosis preoperatively or postoperatively, soft pancreas was defined by surgeons subjectively in this study. In previous reports [21, 22] revealing a relationship between pancreatectomy and CR-POPF, surgeons had judged the pancreas as soft or hard subjectively. Third, the definition of frail varies [16, 20, 32, 33]; thus, our result may be remarkably different than those of previous studies using other definitions. In our report, we adopted the J-CHS criteria, which was a simple frailty assessment tool, and included similar items to the criteria of sarcopenia. Finally, the timing of measuring physical activity and collection of blood samples were not planned and varied among patients. Furthermore, there were patients who underwent nutrition or exercise intervention after being diagnosed frail, and we did not evaluate the effectiveness after these interventions before pancreatectomy. Therefore, future prospective research studies are needed to confirm and evaluate these preliminary findings.

## Conclusion

Frailty may be a useful predictive factor of postoperative complications in patients undergoing pancreaticoduodenectomy. Although many physicians instinctively and subjectively judge whether patients have the physiological reserve to endure operations and postoperative burdens, frailty might be a more effective risk stratification tool to identify potentially high-risk surgical patients undergoing pancreaticoduodenectomy.

## Data Availability

The datasets used and analyzed during the current study are available from the corresponding author on reasonable request.

## Abbreviations

CD: Clavien-Dindo
CI: Confidence interval
CR-POPF: Clinically relevant postoperative pancreatic fistula
J-CHS: Japanese version of the Cardiovascular Health Study
OR: Odds ratio
POPF: Postoperative pancreatic fistula
